# Supplementary material for: Predicting postoperative visual acuity in epiretinal membrane patients and visualization of the contribution of explanatory variables in a machine learning model
Source: PLoS One. 2024 Jul 22;19(7):e0304281. doi: 10.1371/journal.pone.0304281 (PMC11262671; doi:10.1371/journal.pone.0304281)
Supplement: Attachment — Submitted filename: Response to Reviewers.docx [file pone.0304281.s001.docx]

**To the Editors and Reviewers:**

We thank you and the reviewers for the comments and suggestions. We have revised our manuscript accordingly. The revised parts are shown in red font in our revised manuscript. We will be happy to answer any further questions and comments.

Reviewers' comments:
Editorial Office:

1. Please ensure that your manuscript meets PLOS ONE's style requirements, including those for file naming.
**Answer:** Thank you. We have prepared our manuscript to meet PLOS ONE's style requirements.

2. Please note that PLOS ONE has specific guidelines on code sharing for submissions in which author-generated code underpins the findings in the manuscript. In these cases, all author-generated code must be made available without restrictions upon publication of the work. Please review our guidelines at <https://journals.plos.org/plosone/s/materials-and-software-sharing#loc-sharing-code> and ensure that your code is shared in a way that follows best practice and facilitates reproducibility and reuse.

**Answer:** We cannot provide the OCT images used in our experiments as open source because we do not have permission from the Ethics Committee of our hospital. However, we are planning to release the source code used in the experiments. The source code will be made public, but the repository on github has been set to private because the paper is currently under peer review, and we plan to grant DOI after the paper is published.

We will share the details of the source code and execution environment on Google Drive to clarify the current experimental process.

Link to Google Drive: https://drive.google.com/drive/folders/1kQmZbdAD2O5aa51uRiM6FfF5sHzQIG-_?usp=sharing

Link to Github: https://github.com/kokiimai-mieuniv/ERM_pro1

3. Please amend either the title on the online submission form (via Edit Submission) or the title in the manuscript so that they are identical.

**Answer:** Thank you. We have corrected this.

4. We note that Figure 2 in your submission contain copyrighted images. All PLOS content is published under the Creative Commons Attribution License (CC BY 4.0), which means that the manuscript, images, and Supporting Information files will be freely available online, and any third party is permitted to access, download, copy, distribute, and use these materials in any way, even commercially, with proper attribution. For more information, see our copyright guidelines: <http://journals.plos.org/plosone/s/licenses-and-copyright>.

**Answer:** Thank you for confirming my question with more details recently. We have corrected Fig 2 by using an image that has no problem with the Creative Commons Attribution License. In Fig 2 legends, "This figure was created by combining elements from "Human" by Arif Hariyanto, "Eye" by Seaful Muslim, "Data" by AzizGdt, "Brain" by Leo Antho and “Crown” by Kiran Shastry from the Noun Project (https://thenounproject.com). The SHAP logo design is under the MIT License." added (Line 215).

**Reviewer #1**

**Comment 1:** Authors should state the percentage of vitrectomy combined with cataract surgery group and vitrectomy alone. Is there a difference in visual acuity prediction between the two groups?

**Answer:** Thank you for your suggestion. We have considered it and made the necessary updates to the Results section. Specifically, we have included the preoperative and postoperative visual acuity data for both groups, the vitrectomy alone group (1 case) and the vitrectomy combined with cataract surgery group (66 cases). We addressed this in Table1, and the Result section (Line 269). The preoperative visual acuity of the vitrectomy alone group was -0.079. Visual acuity at 1, 3, and 6 months postoperatively was 0.222, 0.0, and -0.079 for both groups, respectively. The study found that the preoperative visual acuity for the group that underwent vitrectomy combined with cataract surgery was 0.172 (standard deviation ± 0.165). Postoperatively, visual acuity at 1, 3, and 6 months was 0.082 (standard deviation ± 0.150), 0.019 (standard deviation ± 0.120), and 0.004 (standard deviation ± 0.128), respectively. It is worth noting that the sample size between the two groups was imbalanced, with only one case in the vitrectomy alone group. Due to the presence of only one case, it was not possible to estimate the variability and, consequently, to assess statistical significance. Therefore, it is difficult to draw any conclusions or make comparisons with the other group.

**Comment 2:** What is the percentage of cases where only ERM was removed or ILM and ERM were removed simultaneously? Is the visual prognosis different in the two groups?

**Answer:** Thank you for your comments. In our study, the total number of patients was 67, and we included the pre- and postoperative visual acuity of the exfoliation of the ERM alone group (24 patients) and the ERM and ILM peeling group (43 patients) in the Results section. The mean preoperative visual acuity was 0.118 (standard deviation ± 0.191) in the ERM peeling group and 0.196 (standard deviation ± 0.138) in the ERM and ILM peeling group, where a statistically significant difference was observed (p = 0.001), indicating that the ERM and ILM peeling group had better preoperative visual acuity. On the other hand, no statistically significant differences were observed between the two groups for visual acuity at 1, 3, and 6 months postoperatively (p values of 0.299, 0.475, and 0.068, respectively). This is added to the Results section in Line 279. We interpret this to mean that both treatment groups achieved similar visual gains at the postoperative time point, suggesting that differences in surgical technique did not significantly affect long-term visual gains.

**Comment 3:** Please consider adding Snellen visual acuity in the method section, as it is difficult to understand the visual acuity in decimal form.

**Answer:** In line 100, we changed 'decimal visual acuity', the only term used in this paper, to 'Snellen visual acuity', as you pointed out.

**Comment 4:** “The exclusion criteria were patients with different degrees of cataracts in both eyes, a decimal best-corrected visual acuity (BCVA) of the fellow eye of <1.0.”Does this mean that there is a cataract affecting vision? Why would Fellow eye vision less than 1.0 be excluded from the study? The meaning of this sentence is difficult to understand.

**Answer:** Many of the eyes in the study (66/67 cases) underwent vitrectomy with combined cataract surgery, and the previously mentioned statement was made in consideration of the exclusion criteria, which would not be affected by cataracts. However, as you pointed out, the text is difficult for readers to understand, so we have changed the exclusion criteria as follows:" The exclusion criteria are 1) cases with different degrees of cataract in both eyes, 2) cases with BCVA of less than 20/20 in the fellow eye,..." in Line 99.

**Comment 5:** An explanation or citation is needed for the ERM stage in the Methods section, not the Results section.

**Answer:** Thank you. The relevant Line225 has been changed as follows:" On the subject of stage classification,..." And we added a citation to the methods section (Line 263).

**Comment 6:** The results section should be shorter. It would be easier for readers to read if some of the results were sent to supplemental data.

**Answer:** In the Results section of the current study, we note the results of two AI models that can explain the clinical predictions that were the goal of our study. The information on the features before feature selection (Tables 1 and 2), the explanatory variables chosen as a result of the subsequent feature selection (Tables 3 and 4), the prediction accuracy (Table 5), and the contribution of the explanatory variables to the prediction in the overall model (Fig 4) and in individual cases (Fig 5), are all described in the results, and any of this We believe that turning to supplements would result in the missing information. However, we acknowledge that your valuable point is most valid and that it is too long.

Specifically, we have removed redundant information from Table 2 (Line 296) and moved the description of VIF to the Methods section (Lines 177-187).

We have made some changes to address the length of the document.

**Comment 7:** Line465. Explanation of SHAP values should be summarized in Methods.

**Answer:** As you indicated, we moved it from the Discussion section to the Methods section (Line 235). Thank you very much.

**Reviewer #2:**

**Comment 1:**  In order to measure these OCT parameters, the authors must exclude those with poor quality OCT images making it difficult to determine the boundary of the different retinal layers. This means that those with DRIL must be excluded. Therefore, stage 4 ERM which represents the most severe form of ERM was excluded in this study. This must be addressed in Methods and Discussion.

**Answer:** Thank you for your suggestion. There were 5 cases of Govetto's stage 4 among the subjects in this study, and we re-checked the image coverage we evaluated for DRIL influence and confirmed that the INL-IPL boundary was identified in all of them, and the area of IRII (A in Fig 1) we used for our features was not affected. We have added this point to our results in Line 296.

**Comment 2:** The authors did not mention about the lens status and if any patients receiving combined vitrectomy and cataract surgery. Theoretically, phakic patients may develop vision-threatening cataract at 6 months postoperatively if they were phakic before surgery. Please add this information.

**Answer:** Thank you for your suggestion. The vitrectomy alone group was 1 of 67 cases in this study, and the preserved lens did not have an increased cataract grade by Emery-Little classification at 6 months postoperatively. We added this information (Line 278).

**Comment 3:** The significance of EIFL for postoperative VA fits clinical intuition. As the authors stated, the means that early intervention is critical in achieving favorable visual outcomes in eyes with an ERM. This should be emphasized and added in the Abstract.

**Answer:** Thank you for the suggestion, we have added one sentence to the abstract at line 57 to mention early intervention.

**Comment 4:** It is difficult for clinicians to understand the meaning of Figure 4 & 5. Please revise the manuscripts to make them more understandable.

**Answer:** Thank you for the suggestion, Fig 4 shows the visualization of the contributions of explanatory variables in the model using Beeswarm Plots, and Fig 5 shows the visualization of the contributions of explanatory variables in representative cases using Waterfall Plot. However, the title of Fig 5 is "Visualization of Contributions of Explanatory Variables in the Model (Waterfall Plot)", which is difficult to distinguish from Fig 4, and we admit that this has caused you some confusion.

Therefore, we have changed the title of Fig 5 to "Visualization of Explanatory Variable Contributions in Individual Cases (Waterfall Plot)".
